# Treatment of the Mouth during the Eruption of the Permanent Teeth

**Published:** 1882-05

**Authors:** 


### ARTICLE VI.

### *Treatment of the Mouth During the Eruption of the Permanent Teeth.*

---

It is generally held to be good doctrine that the deciduous teeth should be retained in the mouth till their successors, the permanent ones, appear; and as thus abstractly recited, the doctrine is undoubtedly sound. The process of nature, when unhindered, needs only to be watched to learn so much. The deciduous teeth erupt, attain their size, do their work, become loose, and fall out to give place to the firmer stronger ones that are to attend man through his chewing days. When these milk-teeth fall, instead of long roots which it was supposed they had, lo! there is seen only a ragged-edged base, and no root at all. Now, it is a fact that the deciduous teeth, in their fullest development, have proportionally longer roots than the permanent ones. Where are they? Absorbed. The pressure upon them of the growing permanent germs, causes absorption. Two results are thus attained. The obstacle, which is the deciduous root, is removed out of the way of its ambitious successors; and an irritation is kept up by means of this pressure, which facilitates the process of development,

Remove the milk tooth while yet its root is of full length, unpushed by its supplanter, and the eruption of the latter is checked, and often delayed for months. Why? The healthful excitement so greatly conducive to its growth is wanting. It is by antagonism we advance. In indolence we stagnate, if not retrograde. The principle runs through nature.

But this normal process may by some means be interrupted or prevented, and a corresponding departure from standard treatment be necessary to counteract the evils which such interruption or prevention would cause. In such a case our so-called "sound doctrine" would be very bad doctrine, and the effect of its application pernicious.

From some cause, to us unknown, the new tooth may be turned from its normal course, and when discovered may pain without or within the proper circle. If this state of things is not corrected, a wide departure from propriety will ensue. The erupting crown of any of the twelve front teeth, in shape a wedge with its base downwards (inwards,) presents its inclined surface to and impinges upon the inclined surface of the root occupying tooth, it also being in shape a wedge with its base upwards (outwards,) and the inevitable result is a growing displacement.

Or the permanent six-year molar and permanent first bicuspid on either side of either jaw may develop to a full size while yet the temporary molar remains firm, and be so shaped and inclined as to overhang, before and behind, this shorter temporary molar, thus locking it in its position firmly. The new second bicuspid coming up to succeed it, makes its way in the track of the dissolving roots of the temporary molar till it reaches the gum margin, where it meets the obstructing wedged crown, which will neither dissolve nor dislodge, when it either remains undeveloped or shoots off in a wrong direceion, causing deformity and injury. This is a condition often overlooked. To correct it, the wedged crown of the temporary molar must at once be removed. Again, a careful examination of the mouth during the period of eruption of the permanent teeth may lead the wise dentist to the conclusion that the upper and lower dental arches, when sufficiently expanded to bear a symmetrical relation to the frame work of the other features, will not accommodate all the teeth which will naturally erupt therein; in other words, that if all the permanent teeth are allowed to take their places, the jaws will be so expanded as to cause disproportion in the features, —the mouth. He will see also, mentally, a crowed arch, teeth forced out of line, contacting surfaces decayed, and an endless need of the dentist's remedial services. This, it may be objected, is too much for a dentist to foresee. I think not. He is supposed to understand the normal development of the region he is educated to treat. He should know, when looking at the nose, cheek bones and chin, what sort of a mouth properly belongs to them. And having closely observed the processes of nature, he should be able to tell what is likely to be the extent of maxillary and alveolar development; how much space there is likely to be in a normally developed alveolar ridge. How, when the first permanent teeth appear, a fair judgement can be formed as to the size of the entire set, and the conclusion quite safely reached whether they will have sufficient room.

If it is concluded that they will not, then the future welfare of that mouth requires that certain teeth be sacrificed. The next question is, which shall they be? While allowing that varying circumstances may vary the decision of this question, I would say, as a rule, the six-year molars. "And why?" queries my sharp reviewer. I will state why. For several reasons:

1st. They are usually the least durable teeth in the mouth.

2d. If extracted before the second molars erupt, the teeth forward of them will naturally, fall back slightly, thus gaining relief from lateral pressure, and the second molars, as they develop, will come gradually forward into the places made vacant, closing up all spaces and making an unbroken masticating surface.

3d. Room being thus at the ends of the alveolar ridge, the *dentes sapientiœ* can erupt more easily, more regularly, less painfully, and in positions where there usefulness will be greatly increased.— *Missouri Dental Journal.*

---

